# A Perceptual Illusion of Empty Space Can Create a Perceptual Illusion of Levitation

**DOI:** 10.1177/2041669519897681

**Published:** 2019-12-30

**Authors:** Heidi Øhrn, Mats Svalebjørg, Steffen Andersen, Anna Edit Ring, Vebjørn Ekroll

**Affiliations:** Department of Psychosocial Science, University of Bergen, Bergen, Norway

**Keywords:** amodal completion, amodal absence, generic view principle, levitation, magic

## Abstract

A recent analysis of magic tricks suggests the existence of a perceptual illusion where the space hidden behind an occluding object is experienced as empty in a strangely compelling way. Here, we show that this illusion of absence is not just a trivial consequence of the lack of retinal stimulation but rather the result of an active process of perceptual construction. The results of a simple experiment show that this perceptual illusion of absence can in turn trigger perceptual processes which generate an immediate perceptual impression of levitation via a percept–percept coupling. This suggests that magical illusions of levitation are partially driven by an immediate perceptual impression of floating in thin air. The perceptual mechanisms underlying the illusion of absence are hitherto unknown, but our results provide support for a potential explanation based on the generic view principle.

The top panels in Figure 1 demonstrate the well-known phenomenon of amodal completion (Kanizsa, 1985; Michotte, Thinès, & Crabbé, 1991; van Lier & Gerbino, 2015). The two aligned fingers in Panel a are experienced as an unnaturally long single finger when the gap between them is hidden behind an occluder (Panel b). Importantly, this compelling illusory impression persists in spite of its absurdity and your better knowledge. The phenomenon of amodal completion challenges naive intuitions about what it means to see: Although our impressions of occluded scene regions obviously refer to parts of objects that do not produce any visual stimulation, they often have properties which are more reminiscent of visual perception than of conscious reasoning and imagery. They often tend to be automatic, immediate, and impervious to conflicting conscious knowledge and beliefs (Ekroll, Mertens, & Wagemans, 2018; Ekroll, Sayim, & Wagemans, 2013; Firestone & Scholl, 2016; Gerbino & Zabai, 2003; Kanizsa, 1985; Michotte et al., 1991; Pylyshyn, 1999). Furthermore, it has been shown that they have functional consequences within the perceptual system (Ekroll, Sayim, Van der Hallen, & Wagemans, 2016; Scherzer & Ekroll, 2009, 2012; Shimojo & Nakayama, 1990), via the so-called percept–percept couplings (Epstein, 1982; Gilchrist, 1977).

**Figure 1.**
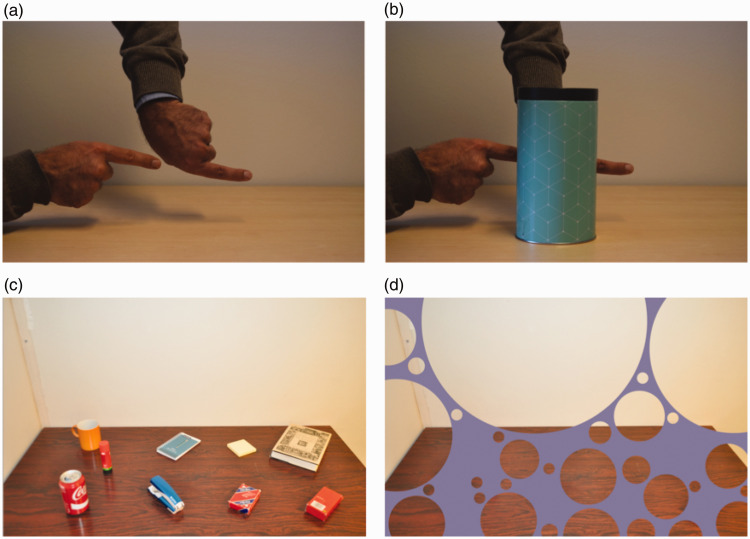
Top row: A demonstration of amodal completion. Two aligned fingers (a) may be compellingly experienced as a single, unnaturally long finger when the gap between them is hidden behind an occluder (b). Bottom row: A demonstration of the illusion of absence. In (d), the objects on the table (c) are occluded by a violet screen in the foreground with round holes in it. Note how difficult it is to imagine that the objects are really hidden behind it. Top row adapted from Ekroll, De Bruyckere, Vanwezemael, and Wagemans (2018), used under CC BY. Bottom row adapted from Ekroll et al. (2017). Copyright (2017) by SAGE Publications. Reprinted with permission.

Extant models of amodal completion appeal to various incarnations of the Gestalt principle of good continuation (Wertheimer, 1923). It is quite obvious, for instance, that the demonstration shown in the top panels of Figure 1 can be explained by appealing to a process that smoothly interpolates the visible contours of the partially occluded objects. Research on amodal completion has demonstrated that it encompasses a very rich set of phenomena (Ekroll et al., 2016, 2018; Gerbino & Zabai, 2003; Koenderink, van Doorn, & Wagemans, 2018; Nanay, 2018; Scherzer & Ekroll, 2015; Tse, 1999; van Lier, 1999; van Lier & Wagemans, 1999), which require more elaborate explanations than simple contour completion. A common feature of all known varieties of amodal completion and corresponding theoretical explanations of it, however, is that they involve visible parts of objects that form the starting point for some kind of perceptual extrapolation, regardless of how elaborate the representations and processes involved may be.

The bottom panels of Figure 1 show a demonstration of an illusion of absence (amodal absence) described in a recent analysis of the role of amodal completion in magic (Ekroll, Sayim, & Wagemans, 2017). Notice the compelling impression that there is nothing lying on the table behind the bubbled occluder in Panel d, although the objects shown in Panel c are actually hidden behind it. It has been argued (Ekroll et al., 2017) that this illusion of absence plays a central role in the art of conjuring. Movie 1 shows an example of a magic trick based on it. When magicians make an object appear out of thin air, it is extremely convenient to produce it from a hiding place close by, which the spectators compellingly, yet erroneously perceive as empty. Obviously, such perceptual voids are just as convenient for making things magically disappear.

At first blush, one might be tempted to conceive of the illusion of absence as a trivial consequence of occlusion. Would it not be only natural that we experience the space behind the occluder as empty given that there is no sensory evidence indicating that there is anything there in this hidden space? But this reasoning does not explain why our brain interprets this absence of evidence as evidence of absence rather than as neutral information indicating uncertainty about what may or may not lie hidden behind the occluder? Thus, the strong feeling of absence is not readily explained by the absence of retinal stimulation per se. At first thought, one might also be tempted to conceive of the illusion of absence as a trivial consequence of amodal completion. Maybe the desk in Figure 1(b) is experienced as empty because the visible parts of the desk are amodally completed behind the occluder? A problem with this reasoning, however, is that the amodal completion of the desk only specifies that there is nothing else in the same depth plane as the desk, but it implies nothing about what may or may not be located in three-dimensional (3D) space *between* the occluder and the desktop.

Thus far, the only evidence for the claim (Ekroll et al., 2017) that the illusion of absence is perceptual in nature are informal observations such as the one shown in the bottom panels of Figure 1. The first aim of this study was to test this claim more rigorously using experimental methods. A potential approach to doing so would of course be to ask subjects to report whether they have any immediate phenomenal experience of the space behind various occluders, and how convinced they are that they experience this space as empty. Reasoning that it may be difficult to obtain reliable reports from naive participants about this, we developed an alternative approach where the illusion of empty space is measured indirectly via a percept–percept coupling (Epstein, 1982). Our approach is based on the observation that there is a logical connection between levitation and empty space. That an object is floating in thin air implies that it is surrounded by empty space on all sides. Thus, if the perceptual system has internalized this logical connection, it may be possible to generate a perceptual illusion of levitation by creating the illusion of empty space.

To anticipate, the results of our experiment strongly suggest that this is indeed the case. In our experiment, we used a simple setup, where a horizontally oriented pencil is balanced on a small vertical pedestal support (see Figures 2 and 3). When this setup is viewed directly (Figure 2(b)), the pencil obviously does not appear to levitate, but rather to rest on the support. By placing a narrow vertical strip in front of the setup such that the vertical support is completely occluded (Figure 2(c) and (d)), but the pencil is only partially occluded in the middle, we aimed to create the perceptual illusion that the support is absent.

**Figure 2.**
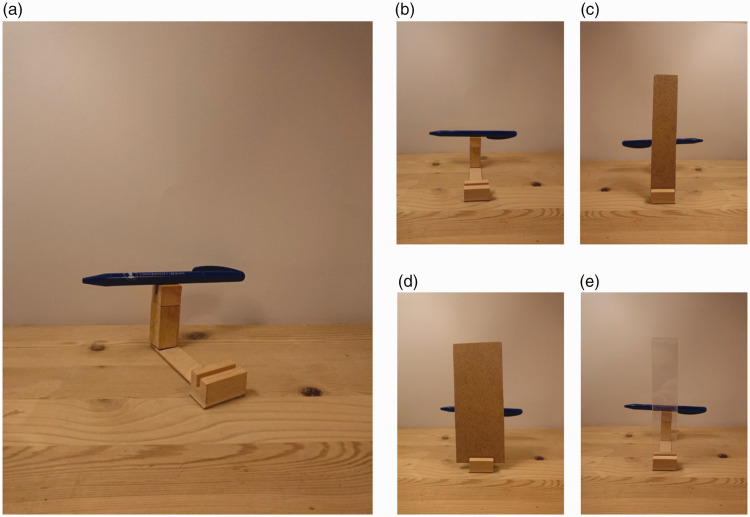
(a) Side view of the setup used in our experiment. A pen rested on a small pedestal support which could be hidden behind a vertical strip. (b–e) Views of the apparatus from the observer’s point of view. (b) Without any occluding strip inserted. (c) With the narrow occluder inserted. (d) With the wide occluder inserted. (e) With the transparent strip inserted (control condition). Note that the illusions of absence and floating do not occur when viewing a photograph of the setup, presumably because a picture does not evoke any compelling sense of a depth difference between the occluder and the pencil (Koenderink, van Doorn, & Kappers, 1994); Vishwanath, 2014). Thus, to experience the effect for oneself, it is better to use some real objects (say a deck of cards as support, and a strip of cardboard as occluder).

**Figure 3.**
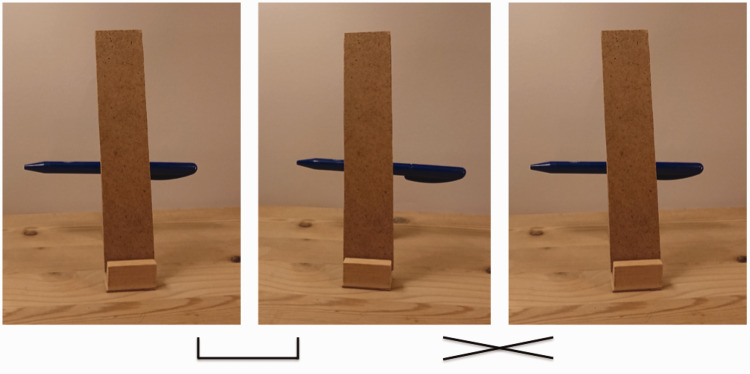
Stereogram of the setup used in the experiment, with the narrow occluder inserted. If you prefer to fuse the images by diverging the eyes, you should fuse the two left images while ignoring the one on the right. If you prefer to converge the eyes, you should fuse the two right images.

Importantly, if observers now have a compelling impression that the pen is floating in thin air, although they know that it is actually resting on the support, we may interpret this as evidence that they experience the space where the support is hidden as empty.

The second aim of our experiment was to test a potential explanation of this illusion of absence. As already explained earlier, extant explanations of amodal completion cannot be applied to this illusion. An explanation appealing to the principle of generic views (Albert, 2001; Freeman, 1994; Koenderink & van Doorn, 1979; Nakayama & Shimojo, 1992), however, which has previously been applied to many other perceptual phenomena, appears viable (Ekroll et al., 2017). According to this principle, the perceptual system excludes interpretations which imply qualitative changes in the retinal image when the viewpoint is changed by a tiny amount. Figure 4 illustrates how predictions about when the illusion of absence can be expected to occur can be derived from the generic view principle. If the occluder is small (blue line in Panel a), a smaller object behind it (red line) may be invisible to both eyes (within the gray region).

**Figure 4.**
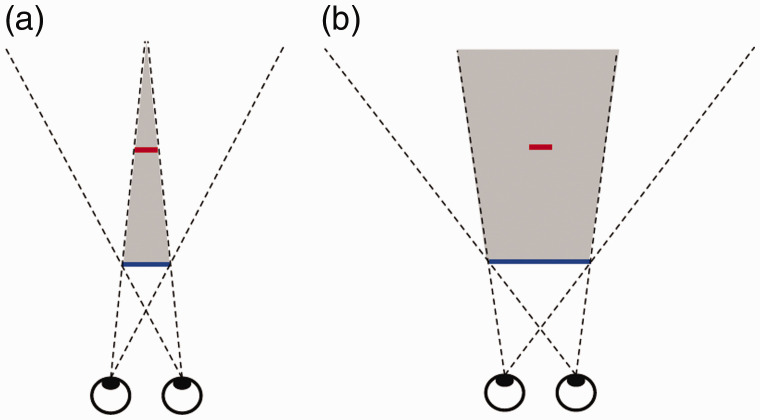
Illustration of how the generic view principle can be applied to the illusion of absence. The shaded area indicates the region of space that is invisible to both eyes due to the occluding object (blue line). (a) With a narrow occluder, a part of the small object (red line) would immediately become visible if the viewpoint changes by a small amount. (b) With a broader occluder, it would remain completely hidden for much larger changes in viewpoint.

If, however, the observer moves slightly to the side, the small object will immediately become visible in one of the eyes, which would involve a qualitative change in the retinal image. Hence, according to the principle of generic views, the perceptual system should exclude the possibility that the white object is present. With a larger occluder (Panel b), however, the occluded object (red line) would remain completely invisible, even when the viewing position changes over a considerable range. Thus, we can derive the general prediction that the illusion of absence should be more likely to occur with a small (or narrow) occluder than with larger (or broader) one.

## Methods

### Apparatus and Stimuli

To investigate our hypothesis, we used a wooden custom-made mounting device where a pen could be placed on a pedestal support in the back, and various occluding strips could be inserted into a slot in front to occlude the view of the pedestal support while the two ends of the pen remained visible (see Figures 2 and 3). We used two opaque occluders of different widths as well as a transparent one to control for potential demand characteristics (Orne, 1962). The narrow opaque screen and the transparent one were both 31 mm wide and the wide screen was 61 mm wide. All of the screens were 153 mm tall.

The mounting device was placed on a plain white table and the participants were seated approximately 70 cm in front of the apparatus. We did not have control over the lighting conditions in the public room where the experiment was carried out, but we took pains to position the device in such a way that any shadows cast by the support were largely invisible.

### Procedure

The experiment was conducted as a semistructured interview, consisting of a free report phase and a rating phase. It was carried out in individual sessions. The participants were randomly assigned to three different groups, which first performed the free report task with one of three different occluder types and then performed ratings with all occluder types in the different orders listed in Table 1. Before the experiment started, the mounting device, the screen, and the pen were shown to the participants. Thus, they were aware that the pen rested on the pedestal support. The participants were told that we were interested in their immediate subjective experience of what they saw, rather than what they know or can deduce. We also emphasized that there were no right or wrong responses.

**Table 1.** Order of the different tasks performed by the three experimental groups.

Group	Time 0 (free report)	Time 1 (rating)	Time 2 (rating)	Time 3 (rating)	
Group A (*n* = 42)	Narrow	Narrow	Wide	Control
Group B (*n* = 41)	Wide	Wide	Control	Narrow
Group C (*n* = 38)	Control	Control	Narrow	Wide

Narrow = narrow opaque screen; Wide = wide opaque screen; Control = transparent screen.

In the first phase of the experiment (the free report phase), one of the three screens was inserted into the instrument while the participant watched. The experimenter then asked the participants to talk freely about what they saw by saying: “When you look at the pen, do you experience anything you find interesting or weird?” The participant’s response to this was recorded on audio for later analysis to establish whether the participant had mentioned that the pen appeared to be floating or not.

After 1 minute, the free report period was terminated and the experimenter immediately went on to ask to what extent the pen appeared to be floating by asking “on a scale from zero to ten, were zero is not at all, and ten is that it completely looks like it, do you think it looks like the pen is floating?”

After both the free report and the direct question phase had been completed with one of the three occluder types, the direct question phase (but not the free report phase) was repeated using the two remaining occluder types. The entire session lasted for about 5 minutes per observer. Approval from the Norwegian Center for Research Data was obtained before the experiment commenced. In accordance with their privacy regulations, the raw audio recordings of the participants’ responses were deleted after they had been classified by our raters (see later). The research was carried out in accordance with the national Guidelines for Research Ethics in the Social Sciences, Humanities, Law and Theology, and written informed consent was obtained from all participants.

### Participants

Participants were recruited from the common area of the student union in Bergen. This resulted in 121 participants varying in age from 18 to 54 years, with a mean of 24 years of age, and a sample with 61 (50.4%) females, and 60 males (49.6%). Participants were not compensated for participation but were free to help themselves to a plate of cookies during the experiment.

Since the experiment was short, we decided to run as many subjects as we could recruit and run in the course of two full working days, although a considerably smaller sample size would have been sufficient for detecting the expected large differences between the experimental conditions and the control condition using traditional statistics at a power level of 95%.

## Results

The data from one participant were omitted because their Norwegian was too poor to understand our questions fully. This left us with data from 121 participants, with an approximately even number of participants in each of the three experimental groups (Table 1). To determine whether the participants had spontaneously made statements to the effect that the pencil appeared to be floating (levitating) or not during the free report period (at Time 0, see Table 1), the audio-recorded responses were analyzed by a research assistant who was unaware of the hypotheses of the study, as well as by author H. Ø. The judgments of the two raters agreed in 114 of the 121 cases (94%). In the following, we base all our analyses on the classifications made by the naïve rater.

**Figure 5.**
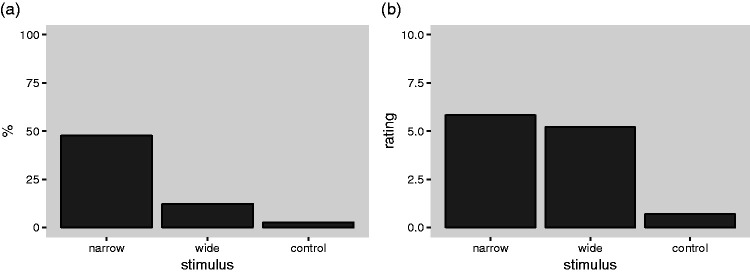
Main results of the experiment. (a) Percentages of the participants who spontaneously mentioned that the pen appeared to levitate in the initial free report phase of the experiment, plotted separately for the three experimental conditions: narrow occluder, wide occluder, and transparent occluder (control condition). (b) Mean ratings of the strength of the impression of floating, pooled across the three groups of participants and the three measurement times (see Table 1).

Figure 5(a) shows the percentages of participants spontaneously mentioning floating during the free-report phase in each of the three conditions. To quantify the strength of the statistical evidence for differences between the conditions, we computed Bayes factors (Dienes, 2011) using the function “contingencyTableBF” from the “BayesFactor” package (Morey, Rouder, Jamil, & Morey, 2015; version 0.9.12-2) for R. Comparing the narrow occluder condition with the control condition, we obtained a Bayes factor of BF_10_ = 24,988, indicating that the data are 24,988 times more likely given the alternative hypothesis of a difference than given the null hypothesis of no difference. According to common terminology (Jarosz & Wiley, 2014), this amount of evidence can be labeled as “decisive.” Comparing the wide occluder condition with the control condition, we obtained a Bayes factor of BF_10_ = 0.47 indicating that the data are 1/0.47 = 2.21 times more likely given the null hypothesis than given the alternative hypothesis of a difference. This amount of evidence can be labeled as “anecdotal” (Jarosz & Wiley, 2014). Finally, comparing the narrow occluder condition with the wide occluder condition, we obtained a Bayes factor of 131, which can be labeled as “decisive” (Jarosz & Wiley, 2014). Thus, there is “decisive” evidence that the narrow occluder condition evokes more floating reports than the control condition, and that the narrow occluder condition evokes more floating reports than the wide occluder condition, but the statistical evidence against a difference between the wide occluder condition and the control condition is merely “anecdotal.”

Figure 5(b) shows the average ratings of the strength of the floating impression for the tree occluder conditions. Here, the ratings have been pooled across groups (and hence also presentation sequence, see Table 1). There is a clear difference between the narrow and wide occluder conditions on the one hand and the control condition on the other hand, but the average ratings in the narrow occluder conditions are only slightly higher than in the wide occluder condition.

**Figure 6.**
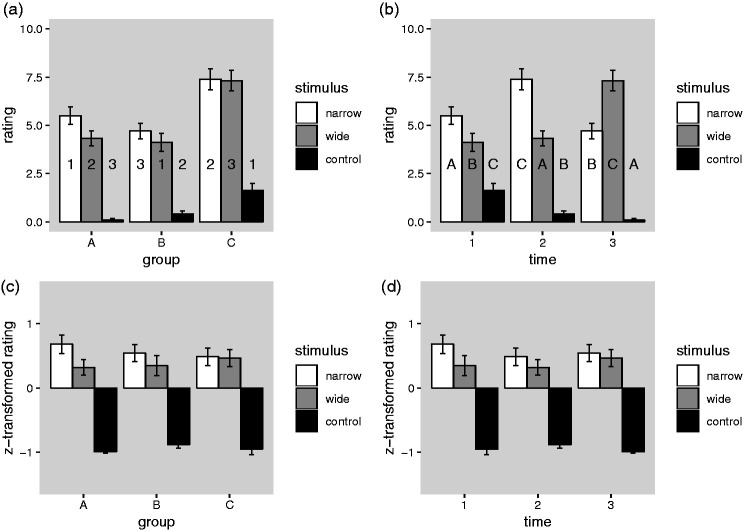
Mean ratings of the strength of the impression of floating, plotted separately for the three stimulus conditions and the three groups of participants (see Table 1). (a) Ratings plotted in a format that facilitates within-group comparisons. (b) Same data, plotted in a format that facilitates between-group comparisons. (c) Same as (a), but after group-wise *z*-tranformation of the raw ratings. Same as (b), but after group-wise *z*-tranformation of the raw ratings. Error bars show ±1 SEM.

In our design (Table 1), each of the three groups of observers rated each of the three stimuli, but in different orders. Thus, we can evaluate our hypotheses based on both between-group and within-group comparisons. Figure 6(a) shows the data in a format that facilitates appreciation of the three possible within-group comparisons, while Figure 6(b) shows the same data in a format that facilitates appreciation of the three possible between-group comparisons. A clear-cut result, which is immediately apparent in these plots, is that the average ratings using the control stimulus are consistently much lower than the average ratings obtained with the two experimental stimuli (the narrow and the wide occluders), irrespective of what comparison is being made. Thus, the evidence for the hypothesis that the opaque occluders have a tendency to evoke illusory floating (and by implication the illusion of absence), which is stimulus-driven rather than a result of a general response bias or demand characteristics, is consistent and clear.

The evidence pertaining to the hypothesis that the illusion of absence is based on perceptual mechanisms working according to the principle of generic views (Albert, 2001), however, is not equally clear-cut. The prediction of this hypothesis is that the ratings obtained with the wide occluder should be lower than those obtained with the narrow occluder. If we first focus our attention on three possible within-group comparisons (Figure 6(a)), we see that this is indeed true at the descriptive level for all three groups, but that these differences are rather small, particularly in Group C. If we focus our attention on the three possible between-group comparisons (Figure 6(b)), however, we see that the differences are in the predicted direction at Time 1 and 2, but in the opposite direction at Time 3. This deviation from the more general pattern of results may be related to the fact that the overall level and range of the ratings is higher in Group C (*M* = 5.45, *SD* = 4.02) than in the two other groups (*M* = 3.31, *SD* = 3.23 for Group A and *M* = 3.08, *SD* = 3.01 for Group B) which can be readily seen in Figure 6(a). If this is taken into account by *z*-transforming the ratings within each group, a much simpler and more coherent picture emerges (Figure 6, bottom panels), where the average values for the narrow occluder are higher than those for the wide occluder for any comparison.

Given that the participants were randomly assigned to the three groups, and each participant performed the experiment in individual sessions, it is implausible that the tendency toward higher ratings in Group C is grounded in anything else than the order in which the three stimuli were presented. A distinguishing feature of the stimulus presentation in Group C is that the control stimulus was presented first. Indeed, before these subjects ever viewed or rated the experimental stimuli, they had already been exposed to the control stimulus twice, namely in the free report session (Time 0, set Table 1) and in the first rating session (Time 1). Considering this, it is not difficult to understand why the participants in Group C tended to use higher ratings. At the presentation of the control stimulus at Time 1, the ratings can be expected to go above zero to the extent that the participants feel that the explicit question about floating put pressure on them to report floating, although they do not really experience it (Orne, 1962). At the presentations of the two subsequent experimental stimuli, several factors may induce the participants to give floating higher ratings than they otherwise would. A striking contrast with the lack of any experience of floating at all at the first two stimulus presentations (Time 0 and Time 1), or the relief of finally being able to report what you are being asked about may bias the ratings upwards. Furthermore, participants who gave nonzero ratings of the control stimulus although they did not really experience any floating may want to “correct” for this when they see the experimental stimuli by giving a correspondingly higher rating afterwards.

To evaluate the statistical evidence for the prediction that the ratings should be higher in the narrow condition than in the wide condition, we analyzed the individual difference scores pooled across all three groups. Here, we obtain a Bayes factor of 13.6 in favor of a difference if we perform a two-sided test, and a Bayes factor of 27.1 in favor of a difference if we perform a one-sided test. Thus, based on the within-subject comparisons, the overall statistical evidence for the predicted difference is “strong.”

**Figure 7.**
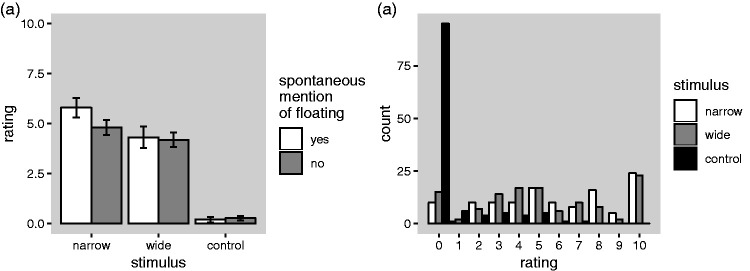
(a) Floating ratings plotted separately for participants who did and did not spontaneously mention floating in the initial free report phase of the experiment. (b) Distribution of the floating ratings for each of the three stimulus conditions.

We now consider the relationship between the spontaneous reports of floating (Figure 5(a)) and the floating ratings (Figures 5(a) and 6). Although a sizable proportion of the participants spontaneously reported floating, particularly in the narrow occluder condition, the majority did not (see Figure 5(a)). This could mean that some people have a perceptual experience of floating, while others do not, but it could also mean that everybody perceive floating, but that some participants fail to report it spontaneously, for instance, because they are reluctant to report on a patently weird experience that contradicts their factual knowledge. Based on the former hypothesis, one would expect that people who spontaneously report floating should give higher ratings of the floating impression than people who did not. As can be seen in Figure 7(a), however, which shows the average floating ratings plotted separately for these two groups of participants, the ratings are very similar for the two groups in all of the three stimulus conditions (note that we only consider participants in Groups A and B here, because there is no reason to expect that participants in Group C, who were presented with the control condition in the free report phase, should spontaneously report floating). A Bayesian mixed-model analysis of variance (Morey et al., 2015) including stimulus condition as an additional fixed factor and subject as a random factor provided “substantial” (Jarosz & Wiley, 2014) evidence against a main effect of the “spontaneous mention of floating” (yes or no; B_10_ = 0.30).

An interesting feature of the floating ratings that is not apparent in the previous plots (which only show averages) is that while the distribution is sharply peaked at 0 in the control condition, the distributions in the narrow and the wide conditions extend widely over the available response range (Figure 7(b)).

### Additional Results From a Similar Pilot Experiment

For completeness, we should mention that the results of a similar pilot study (Andersen, Ring, Svalebjørg, & Ekroll, 2017) in which we only used the narrow occluder and the control condition broadly agree with the present findings. The percentage of observers who spontaneously reported floating in the narrow condition was somewhat lower in the pilot experiment (33%, 20 out of 60 participants) than in the present experiment (48%, 20 out of 42 participants). In the pilot experiment, we only collected ratings from those subjects that spontaneously reported floating. Because nobody spontaneously reported floating in the control condition, we only had rating data from the narrow occluder condition. The average rating was 4.75 (*n* = 20), compared to 5.83 (*n* = 121) in the present experiment.

## Discussion

Our results show that an object resting on a support can be perceived as “magically” floating in thin air if an object in the foreground occludes the view of the support. Importantly, this illusion persisted in spite of the participants’ explicit knowledge that the object was actually resting on the support. Given that such a persistence in spite of conflicting conscious knowledge is considered a hallmark of perceptual processing (Ekroll et al., 2013; Firestone & Scholl, 2016; Leslie, 1988; Pylyshyn, 1999), this strongly suggests that the illusion is a product of hitherto unknown perceptual processes.

It is difficult to explain our findings in any other way than by assuming (a) that the perceptual system creates an illusion of empty space behind the strip and (b) that there is a perceptual attribute of “floating in thin air,” which is (c) linked to the perception of empty space via a percept–percept coupling (Ekroll et al., 2016; Epstein, 1982; Gilchrist, 1977). Thus, our results not only provide evidence that the illusion of absence is based on perceptual processing but also that the impression that something is “floating in thin air” can be a purely perceptual attribute which is independent of conscious knowledge and reasoning.

The illusion of absence is similar to amodal completion in the sense that it refers to occluded regions of a visual scene. A further similarity supported by the present findings is that both of these phenomena are due to perceptual processes. Indeed, one interesting way to think about the illusion of absence that highlights its similarity to amodal completion even further is to conceive of it as amodal volume completion (Tse, 1999; van Lier, 1999) of the empty 3D space to the side of the occluder. At a descriptive, phenomenological level, it is tempting to conceive of the empty space surrounding objects in a 3D visual scene as a 3D analogue of the concept of “ground” (Rubin, 1915) in two-dimensional pictorial figure-ground perception. Thus, in much the same way as the ground is experienced as extending behind the figure in pictorial two-dimensional displays (which Nelson & Palmer, 2007, consider “a weak form of amodal completion”), one could say that the 3D empty space to the side of an occluder is experienced as extending behind (or around) it. Indeed, in their discussion of some very interesting cases of “amodal completion without cover,” Michotte et al. (1991, pp.161–163) seem to have had such an idea in mind, when they noted that it “is perhaps possible, if one extends the notion of amodal completion, to apply it to the interpretation of the way in which a perceptual field is structured—so-called ‘empty space’, separation of objects in space, distance between them, etc.” (p. 163).

Although amodal completion and the illusion of absence thus can be regarded as strikingly similar or at least analogous at the level of phenomenological description and in terms of their cognitive impenetrability, it is difficult to see how the theoretical underlying mechanisms or processes thought to be responsible for amodal completion can account for the illusion of absence. All extant explanations of amodal completion appeal to some kind of extrapolation of the visible parts of objects, but the illusion of absence simply does not refer to objects with visible parts. Thus, depending on whether amodal completion and the illusion of absence are based on common or separate underlying mechanisms, we either have to revise current explanations of amodal completion or postulate separate new explanations for the illusion of absence.

In our experiment, we tested a candidate separate explanation for the illusion of absence appealing to the principle of generic views (Albert, 2001). The results from the free report phase provided quite strong support for this prediction. The evidence from the ratings in the second part of the experiment is weaker, but points in the same direction and is still in a range, which is commonly labeled as “strong evidence” (Jarosz & Wiley, 2014). Further experimental work is required in order to draw definitive conclusions about the ultimate merit of this explanation. Experiments where the size of the occluder is varied over a broader range would be of great interest. This could be done both with the current paradigm and with a paradigm based on self-occlusion (as in Movie 2).

A potential confound in our investigation of the influence of the occluder width is that a broader occluding screen necessarily entails a corresponding increase in the gap between the visible parts of the pen (and hence reduce the support ratio, see Gerbino & Fantoni, 2006), which may be expected to make the amodal completion of the pen less compelling. It is conceivable that such a reduced strength in the amodal completion of the pen could influence the experience of floating. Weaker amodal completion of the visible parts of the pen could make it appear more like two unconnected parts than a single object. If the illusion that the support is absent is strong and clear, this should be irrelevant, because absence of the support would imply that the parts of the pen are floating, whether they are connected or not. Conversely, though, if the illusion that the support is absent is weak or absent, this could increase the tendency to experience the perceptually unconnected parts as magically floating due to a lack of perceived stability/balance (Cholewiak, Fleming, & Singh, 2013; Ekroll & Wagemans, 2016): A whole pen can rest in balance on the support in the middle, but separate parts cannot. Thus, it is conceivable that this confound creates a tendency to experience floating even when there is no illusion of absence. Since this would be more likely to occur with the wide occluder, it would create a bias working against the prediction of the generic views hypothesis. It is more difficult to conceive of a reason how this confound could create a bias that would work in the same direction as the prediction of the generic views hypothesis. Thus, this potential confound could mean that our data underestimate the actual influence of occluder width on the illusion of absence.

### Individual Differences: Variations in Perception or Variations in the Weight Given to Conflicting Knowledge?

Although the evidence for the floating illusion is very strong at the aggregate level, it is notable that only about half of the participants spontaneously mentioned it in the most potent stimulus condition (narrow occluder, see Figure 5(a)). Relatedly, it is also notable that the average ratings of the strength of the floating impressions are only intermediate (Figure 5(a)) and that the ratings in the experimental conditions are very broadly distributed across the response range (Figure 7(b)). This may reflect individual differences in the perceptual susceptibility to the illusion, but it may equally well reflect different individual tendencies to let the conflicting explicit knowledge that the pen was actually not levitating influence the responses. Although we explicitly asked the participants to report their experiences rather than their factual knowledge, it may be difficult for participants to distinguish what they know from their immediate perceptual experience. Some participants may have been reluctant to volunteer information about strange experiences which are patently at odds with reality or to give high ratings of floating because they did not want to come across as airy-fairy or gullible.

The observation that the average ratings were similar for subjects who did and subjects who did not spontaneously reported floating in the free report phase suggests that the inclination to spontaneously report floating does not reflect genuine individual differences in the perceptual susceptibility to the floating illusion.

### Implications for Illusions of Levitation in Magic

Illusions of levitation are routinely used by magicians (Kuhn, Caffaratti, Teszka, & Rensink, 2014; Lamont, 2017; Leddington, 2016; Ottaviani & Johnson, 2007). The present findings suggest that a major source of the illusion is perceptual in nature. Although a support may be hidden behind the floating object (which is actually quite frequently the case, see e.g., Ottaviani & Johnson, 2007), the spectators tend not to think of this possibility due to the powerful visual illusion of floating, which in turn is linked to the visual illusion that the space behind the putatively floating object is empty. Movie 2 gives a demonstration of this.

## Conclusions

Our findings strongly suggest that the illusion of absence is not just a trivial consequence of the lack of retinal stimulation, but rather the result of an active process of perceptual construction. Furthermore, they also strongly suggest that the illusion of absence can trigger perceptual processes, which generate an immediate perceptual impression of levitation via a percept–percept coupling. Our findings also provide preliminary support for an explanation of the illusion of absence based on the principle of generic views (Albert, 2001).

## Supplementary Material

Supplementary material

Supplementary material
